# Reference values for N-terminal Pro-brain natriuretic peptide in premature infants during their first weeks of life

**DOI:** 10.1007/s00431-020-03853-8

**Published:** 2020-11-03

**Authors:** Agnes-Sophie Fritz, Titus Keller, Angela Kribs, Christoph Hünseler

**Affiliations:** Neonatal Intensive Care Unit, University Hospital in Cologne, Kerpener Str. 34, 50937 Cologne, Germany

**Keywords:** N-terminal pro-brain natriuretic peptide, Preterm infant, Reference values

## Abstract

The aim of our study was to observe the temporal distribution of serum N-terminal pro-brain natriuretic peptide (NT-proBNP) in premature infants of ≤ 31 weeks of gestational age (GA) during the first weeks of life. NT-proBNP values of 118 preterm infants born ≤ 31 weeks GA were determined during the first week of life, after 4 ± 1 weeks of life, and at a corrected GA of 36 ± 2 weeks. Infants were divided into two groups: those without relevant complications and those with complications related to prematurity. NT-proBNP values of infants without complications define our exploratory reference values. The Median NT-proBNP level of these infants was 1896 ng/l (*n* = 27, interquartile range (IQR): 1277–5200) during the first week of life, 463 ng/l (*n* = 26, IQR: 364–704) at 4 ± 1 weeks of life, and 824 ng/l (*n* = 33, IQR: 714–1233) at a corrected GA of 36 ± 2 weeks. Infants born < 28 + 0 weeks GA had significantly higher NT-proBNP values (*n* = 9, median: 5200, IQR: 1750–8972) than infants born ≥ 28 + 0–31 weeks GA (*n* = 18, median: 1528, IQR: 838–3052; *p* = 0.017). Growth restriction or PDA status could not account for the difference in NT-proBNP values between GA groups.

*Conclusions*: The results of our observational and cross-sectional study describe exploratory reference values for NT-proBNP levels in preterm infants of ≤ 31 weeks GA according to postnatal age. NT-proBNP levels during the first week of life are high and widely distributed in preterm infants and decrease subsequently to reach a distinctly lower and stable plateau at around 1 month of life. Our results suggest an influence of GA on NT-proBNP values in the first week of life.

**Table:** 

**What is Known:** *• Several complications related to prematurity, e.g., hemodynamically significant PDA, pulmonary hypertension, bronchopulmonary dysplasia, and retinopathy of prematurity, have been associated with a temporary rise in NT-proBNP values in preterm infants during their first weeks of life.* **What is New:** *• This observational study provides reference values for NT-proBNP levels of very and extremely preterm infants during their first weeks of life.* *• In premature infants without complications, NT-proBNP values during their first week of life depend on gestational age at birth*.

**Supplementary Information:**

The online version contains supplementary material available at 10.1007/s00431-020-03853-8.

## Introduction

Brain natriuretic peptide (BNP) consists of 32 amino acids, is predominantly produced in cardiomyocytes of the ventricles of the heart, and is secreted in response to cardiac stress, e.g., to volume expansion or pressure overload to regulate blood pressure by increasing diuresis, natriuresis, and vasodilation. Cardiomyocytes secrete an inactive precursor proBNP which is then cleaved into the active BNP and the inactive N-terminal pro BNP (NT-proBNP) [1]. NT-proBNP shows more stability in vitro and has a longer half-life than BNP (120 min vs. 22 min) [2]. NT-proBNP and BNP are renally excreted and particularly NT-proBNP plasma level is known to increase with decreased kidney function [3, 4]. BNP is an established marker of congestive heart failure in adults [5].

NT-proBNP is associated with different diseases of prematurity; increased NT-proBNP and BNP levels have been found in infants with hemodynamically significant patent ductus arteriosus (hsPDA) [6–9], bronchopulmonary dysplasia (BPD) [10–15], pulmonary hypertension (PH) [16–22], retinopathy of prematurity (ROP) [23, 24], inflammation or sepsis [25–27], and congenital heart diseases [28, 29], but published data remain inconclusive.

Currently, there are no established and validated normal range of values for very and extremely preterm infants and the results seem to vary depending on gestational age (GA), the postnatal age of testing, the diagnostic test kits used, and the underlying conditions of the patients. The aim of this observational study is to describe exploratory reference values of serum NT-pro BNP in premature infants of ≤ 31 weeks of GA during their first weeks of life.

## Materials and methods

In this retrospective observational analysis data, 118 preterm infants ≤ 31 weeks GA treated between October 1, 2017, and June 30, 2019, in the Neonatal Intensive Care Unit (NICU) of the Children’s Hospital of the University Hospital in Cologne, Germany, were included. The study was approved by the Bioethics Committee of the University of Cologne, and parental consent was not required due to the routine collection of NT-proBNP. Determination of NT-proBNP levels in any preterm infant born < 32 weeks GA is part of routine blood collections at our institution, mainly to detect infants at risk for the development of PH and BPD. NT-proBNP measurements are routinely done in the first week of life, around day 28 of life, and when infants reach a corrected GA of 36 weeks. The last two sampling times were chosen according to the critical periods defining BPD.

Due to practical and clinical conditions timing of NT-proBNP, measurements partly differ from the institutional protocol and the following three sampling times were identified: day 2 to 11 of life (in the following called: first week of life), day 18 to 36 of life (4 ± 1 weeks of life), and when the preterm infants reached a corrected GA of 34 + 3 to 40 + 3 weeks (corrected GA of 36 ± 2 weeks). If a preterm infant had multiple samples drawn during the same sampling time, the higher NT-proBNP value was chosen. Missing values emerged randomly or were due to a condition of the neonate that did not allow blood sampling, a potential source of bias.

**Fig. 1:**
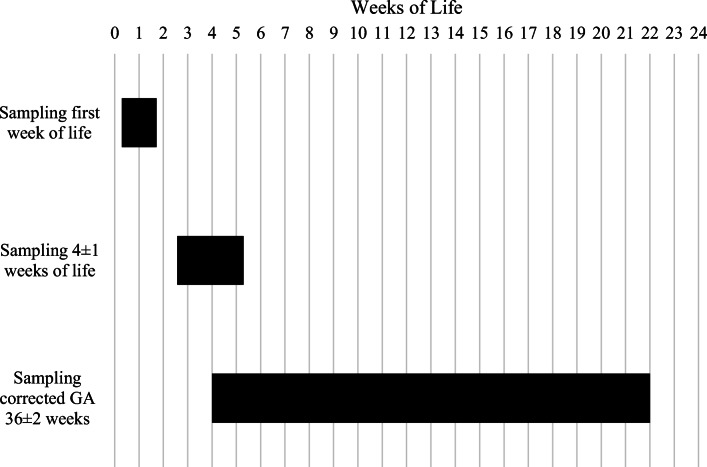
Sampling times according to weeks of life

Figure 1 shows the different sampling times according to weeks of life. The samplings at 4 ± 1 weeks of life and at a corrected GA of 36 ± 2 weeks partly overlap due to the possibility that in less premature infants, the chronological age of around 4 weeks overlaps with the corrected GA of 36 weeks. Samplings were included if they could be assigned to one sampling time.

We stratified the cohort into infants without relevant complications and those with complications related to prematurity. If one infant had any of the following complications at any time, all its NT-proBNP values were analyzed in the group with complications. The following criteria define the different complications:

I. Infection: elevated laboratory values (interleukin-6 > 50 ng/l, C-reactive protein (CRP) > 5 mg/l, leucocytes > 20,000/μl or left shift) or clinical signs of infection with subsequent antibiotic treatment
II. Hemodynamically significant PDA: two of the following criteria were observed by echocardiography: (1) diameter of the ductus arteriosus: ≥ 2.0 mm, (2) degree of pulmonary overflow: left atrial to aortic root ratio (La:Ao) ≥ 1.4, (3) retrograde descending aortic flow, (4) organ blood flow: low antegrade flow in systole or diastole or absent/reversed diastolic flow
III. BPD: definition according to Jobe [30]
IV. Pulmonary hypertension: defined by (1) elevated tricuspid regurgitation jet velocity with an estimation of RV pressure of more than 25 mmHg (depending on systemic pressure: > 50% of systemic pressure), (2) interventricular septum-configuration: flat or bowed to the left, (3) right to left or bidirectional shunting at the patent foramen ovale or PDA [31]
V. ROP stage 2 with plus disease or higher according to the international definition of ROP [32]
VI. Intraventricular hemorrhage (IVH) grade 2 or higher according to Papile [33]
VII. Pulmonary complications, e.g., pneumothorax or pulmonary interstitial emphysema
VIII. Intestinal complications, e.g., all stages of necrotizing enterocolitis (NEC) according to the modified Bell staging criteria [34], focal intestinal perforation (FIP), or gastric perforation.

Infants with major congenital heart diseases, chromosomal abnormalities, or kidney diseases were excluded.

Additionally, we tested the influence of each complication on NT-proBNP values separately comparing the NT-proBNP values of infants with and without the respective complication.

In a second step, we investigated if our exploratory NT-proBNP reference values depend on GA at birth and divided the infants according to the World Health Organization’s (WHO) definition of extremely (born < 28 weeks GA) and very preterm infants (born between 28 and 32 weeks GA).

Originally based on a longitudinal study design, the composition of groups differs over time resulting that the study holds the characteristics of a cross-sectional study.

Arterial, central venous, peripheral venous, or capillary blood samples were analyzed on the Cobas E801 analyzer using the Elecsys proBNP II method (Roche Diagnostics). Different types of blood sampling presumably lead to comparable NT-proBNP values [35, 36]. The measuring range for the used method is 5–35,000 ng/l. Values above the upper measuring limit are reported as 35,000 ng/l or up to 70,000 ng/l for 2-fold diluted samples. The cut-off value for the diagnosis of heart failure in adults is 125 ng/l for the used test kit. According to Roche Diagnostics considering concentration-dependent scattering, the intra-assay coefficient of variation for human serum varied between 1.5 and 9.2%, and the inter-assay coefficient of variation for human serum between 2.6 and 12.5% [37]. High levels of hemoglobin, triglycerides, or bilirubin do not seem to interfere with NT-proBNP analysis [38]. Different NT-proBNP assays show poor concordance. Therefore, assay-specific reference and cut-off values must be considered when interpreting NT-proBNP levels and patients should be monitored using the same assay [39, 40].

## Statistical analysis

Statistical analysis was performed using the Statistical Package for Social Sciences, v24 (IBM SPSS, Chicago, Ill., USA). The Mann-Whitney *U* test was used to compare NT-proBNP concentrations between groups, and the Friedman test to evaluate changes in NT-proBNP levels over time. A two-sided *p* value of < 0.05 was considered statistically significant.

## Results

### Patient cohort

Characteristics and postnatal course of the 118 patients, including the prevalence of prematurity-related complications in our cohort, are described in Table 1. The median GA at birth was 26 + 5 weeks and the median birth weight is 900 g. Nine preterm neonates were small for gestational age (SGA; defined by a birth weight < 3rd percentile). The median 5′ Apgar score was 7 and the median CRIB-II score 5. A total of 108 patients were delivered by C-section and mothers of 90 infants had prenatal steroids administered. One patient died after 5 days because of severe IVH.

**Table 1:** Characteristics and postnatal course of patients

Characteristics	All infants: *n* = 118 number (percentage) or median (IQR)
Gender, male	69 (59%)
Gestational age at birth (weeks)	26 + 5 (24 + 5–29 + 2)
Birth weight (g)	900 (630–1220)
SGA (birth weight < 3rd percentile)	9 (8%)
5′ Apgar score	7 (7–8)
CRIB-II score	5 (2–9)
C-section	108 (92%)
Prenatal steroids	90 (76%)
Complete	67 (57%)
Incomplete	23 (19%)
Postnatal course	
Chorioamnionitis	12 (10%)
Infection	35 (30%)
PDA	94 (80%)
Non-hemodynamically significant	76 (64%)
Hemodynamically significant	18 (15%)
Pulmonary hypertension [33]	24 (20%)
BPD [32]	37 (31%)
Mild	26 (22%)
Moderate	6 (5%)
Severe	5 (4%)
ROP [34]	101 (86%)
Stage 1	79 (67%)
Stage 2	12 (10%)
Stage 3	10 (8%)
IVH [35]	37 (31%)
Grade 1	23 (19%)
Grade 2	3 (3%)
Grade 3	11 (9%)
Intestinal complications	15 (13%)
NEC [36]	4 (3%)
FIP	9 (8%)
Gastric perforation	2 (2%)
Pulmonary complications (pneumothorax, pulmonary interstitial emphysema)	10 (8%)
Duration of ventilation (mechanical ventilation and CPAP) (hours)	835 (231–1564)
Duration of oxygen supply > 21% (days)	3 (1–46)

#### A. Exploratory reference values for NT-proBNP in preterm infants ≤ 31 weeks GA during the first weeks of life

**Fig. 2:**
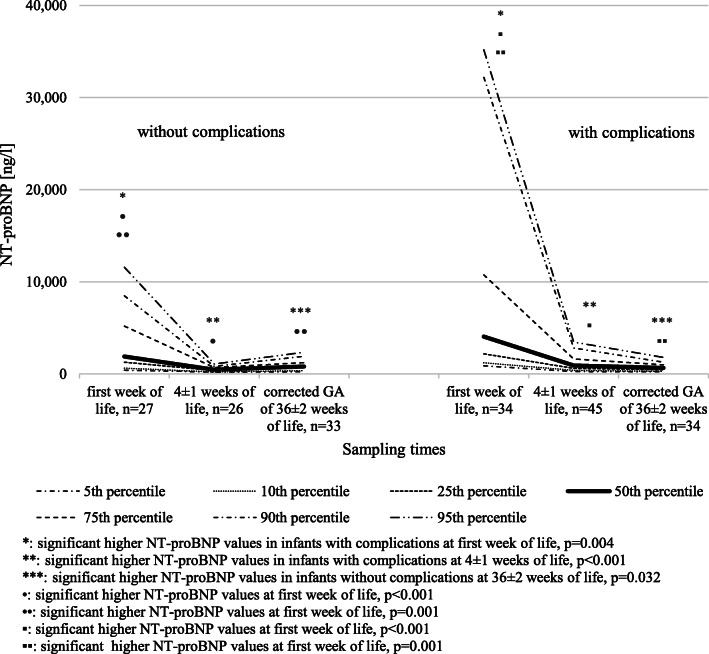
Nomograms showing the 5th, 10th, 25th, 50th, 75th, 90th, and 95th percentile for NT-proBNP values in ng/l in preterm neonates born ≤ 31 weeks GA over the first weeks of life. NT-proBNP values for preterm infants without relevant complications are presented on the left side, with relevant complications on the right side

NT-proBNP values of infants without relevant complications related to prematurity define our exploratory reference values. Comparing NT-proBNP values of infants without major complications to those of infants with complications using the Mann-Whitney *U* test, NT-proBNP values of preterm infants with relevant complications were significantly higher during the first week of life (*n* = 27, median: 1896, IQR: 1277–5200 vs. *n* = 34, median: 4058, IQR: 2189–10,751; *p* = 0.004) and at 4 ± 1 weeks of life (*n* = 26, median: 463, IQR: 364–704 vs. *n* = 45, median: 920, IQR: 601–1630; *p* < 0.001). At a corrected GA of 36 ± 2 weeks, preterm infants without relevant complications had significantly higher NT-proBNP values compared to the group with complications (*n* = 33, median: 824, IQR: 714–1232 vs. *n* = 34, median: 671, IQR: 449–989; *p* = 0.032). The nomogram in Fig. 2 describes the 5th, 10th, 25th, 50th, 75th, 90th, and 95th percentile for NT-proBNP values of preterm infants without and with complications over the first weeks of life. Tables 2 and 3 (see Online Supplement) show the corresponding values. We separately analyzed the NT-proBNP values of the infants displaying the single complications (infection, hsPDA, BPD, PH, ROP, IVH, pulmonary and intestinal complications) and compared them to the NT-proBNP values of infants without the respective complication. We could demonstrate significantly higher NT-proBNP values in infants with hsPDA (first week of life, *p* = 0.034), BPD (first week of life, *p* = 0.021; 4 ± 1 weeks of life, *p* < 0.001), PH (4 ± 1 weeks of life, *p* < 0.001), ROP stage 2 with plus disease or higher (4 ± 1 weeks of life, *p* = 0.007), infection (first week of life, *p* = 0.02, 4 ± 1 weeks of life, *p* = 0.032), IVH grade 2 or higher (first week of life, *p* = 0.014; 4 ± 1 weeks of life, *p* = 0.001), and intestinal complications like NEC, FIP, or gastric perforation (first week of life, *p* = 0.029; 4 ± 1 weeks of life, *p* = 0.007). The exact values are given in Tables 5–28 in the Online Supplement.

#### B. Dependency of exploratory NT-proBNP reference values on the gestational age at birth

Infants without relevant complications that define our exploratory reference values were divided into two groups depending on GA at birth: extremely preterm infants (< 28 + 0 weeks GA) and very preterm infants (≥ 28 + 0–31 weeks GA). During the first week of life, NT-proBNP values of the extremely preterm infants (*n* = 9, median: 5200, IQR: 1750–8972) were significantly higher compared to the values of the very preterm infants (*n* = 18, median: 1528, IQR: 838–3052; *p* = 0.017). At 4 ± 1 weeks of life and at a corrected GA of 36 ± 2 weeks, no statistically significant differences in NT-proBNP values were seen between the different GA groups (*p* = 0.421, *p* = 0.703).

During the first week of life, no infant of the two different GA groups was SGA. Eight (89%) infants of the extremely preterm infants had a nhsPDA, in comparison to 8 (44%) of the very preterm infants. NT-proBNP values of infants with different PDA status (no vs. nhsPDA) did not differ significantly within the groups of extremely and very preterm infants (*p* = 0.222, *p* = 0.101). In multiple linear regression analysis (*p* = 0.051, *R*^2^ = 0.219), GA at birth but not PDA status proved to have a significant correlation with NT-proBNP values during the first week of life (*p* = 0.038).

#### C. Development of NT-proBNP values over time

**Fig. 3:**
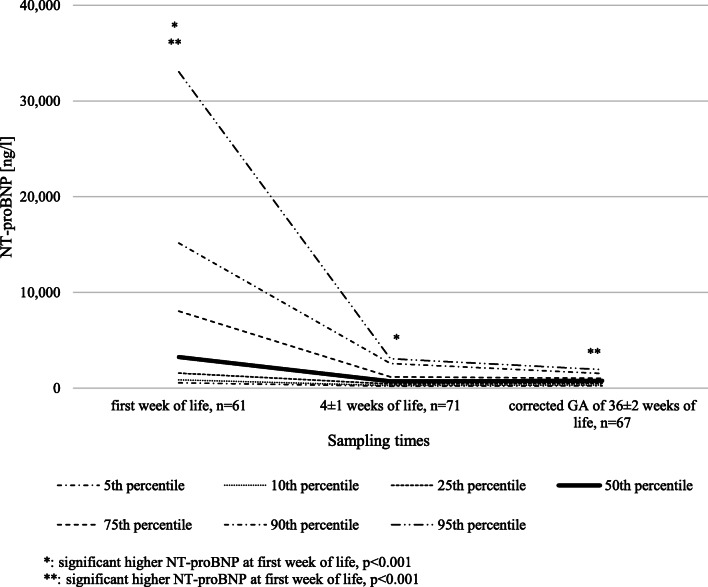
Nomogram showing the 5th, 10th, 25th, 50th, 75th, 90th, and 95th percentile for NT-proBNP values in ng/l in preterm neonates born < 31 weeks GA over the first weeks of life

NT-proBNP values showed a very wide range of distribution (minimum: 148 ng/l, maximum: 39,340 ng/l) with the highest values during the first week of life. Values then decreased to a distinctly lower and stable plateau at 4 ± 1 weeks of life and at a corrected GA of 36 ± 2 weeks (Fig. 3, Table 4 in Online Supplement).

Comparing the NT-proBNP values within the infants without and with complications using the Friedman test, NT-proBNP values were significantly higher during the first week of life than at 4 ± 1 weeks of life and at a corrected GA of at 36 ± 2 weeks. Between NT-proBNP values of 4 ± 1 weeks of life and a corrected GA of at 36 ± 2 weeks, no statistical difference was seen (Fig. 2).

## Discussion

The current study investigated the temporal distribution of NT-proBNP values and describes exploratory reference values in premature infants born ≤ 31 weeks GA.

Most studies investigating NT-proBNP values in preterm infants have focused on the presence of a defined complication of prematurity and compared the NT-proBNP values to values of infants not displaying this complication—irrespective of other possible confounding complications. We aimed to exclude all conditions that could possibly influence NT-proBNP values. For some of these conditions, there is strong, study-based evidence and a direct pathophysiological link on how they influence NT-proBNP levels in preterm neonates, among them hsPDA [6–9], BPD [10–15], and PH [16–22]. For others, e.g., ROP and infection, the pathophysiological link to NT-proBNP is not as clear. Still, high urinary NT-proBNP levels and subsequent ROP development have been described, in a multicenter trial with 1000 patients [23]. A correlation between NT-proBNP and inflammation or sepsis observed in some studies is explained in part by acute ventricular dysfunction in septic patients [26] and in part by markers of inflammation that may selectively influence BNP gene expression [25, 27]. For some conditions, including intestinal complications (NEC, FIP, gastric perforation), pulmonary complications (pneumothorax, PIE), and IVH, data regarding the relationship between NT-proBNP plasma levels and the complications are still lacking. Nevertheless, these conditions are often accompanied by hemodynamic changes due to elevated intra-abdominal or intra-thoracic pressures, volume depletion, and inflammation that are likely to affect NT-proBNP levels.

Indeed, in an internal analysis, we were able to show significant higher NT-proBNP values for most of our complications. However, further analysis, including multivariate regression analysis, can potentially shed more light on these relationships.

NT-proBNP values of infants with complications were significantly higher during the first week of life and at 4 ± 1 weeks of life compared to those without complications. NT-proBNP values at a corrected GA of 36 ± 2 weeks stand out as infants without complications tended to have higher values compared to those with complications. With increasing postnatal age, NT-proBNP values decline after birth. Infants with complications and a more likely lower GA at birth had a higher chronological age at 36 weeks corrected GA compared to the reference group which may represent in lower NT-proBNP levels. In contrast, in infants without major complications, NT-proBNP values increased from 4 ± 1 weeks of life to the corrected GA of 36 ± 2 weeks. Interestingly, Harris et al. studied NT-proBNP levels in a comparable cohort of preterm infants and described a similar increase in values of the control group at a corrected GA of 36 weeks [6]. On the other hand, Nir described a continuous decline in values with increasing chronological age in healthy term neonates [41]. It is important to emphasize that the median GA of our cohort is 26 + 5 weeks and the median birth weight is 900 g. Hence, our cohort is shifted to extremely premature infants complicating the comparison to other epidemiological studies in premature infants.

NT-proBNP levels of our study as in others [6, 41] peak in the first days after birth. Values then decrease to lower levels with narrow ranges, which are though much higher than the normal range known from healthy adults. The peak in the first days of life is most likely due to the significant circulatory changes during transition from fetal to neonatal circulation. During this period, the individual trends in one patient’s values may be more useful than generally defined reference values.

Exploratory reference values in infants < 28 weeks GA were significantly higher than in those of 28–31 weeks GA at birth. We further investigated if comorbidities could account for the observed difference, as several studies have previously assumed [6, 42, 43]. Neither growth restriction nor different PDA status (no vs. nhsPDA) could account for the observed difference. Further studies are required to investigate the dependency of NT-proBNP values on the GA at birth which is complicated by the fact that only a small number of extremely preterm infants remain without relevant comorbidities in the early postnatal life.

Future studies that aim to help define NT-proBNP reference values should be designed as prospective studies, define more strictly sampling times, and evaluate NT-proBNP values longitudinally with an increased number of patients to gain more statistical power.

## Study limitations

As the composition of patients within a group differs over time, the study carries characteristics of a cross-sectional study. Blood collection differed from the time points defined in the NICU standard which may have had an impact especially on the results during the first week of life when significant changes may occur daily. Another study limitation is the retrospective nature and the resulting limitation of standardization. The moderate sample size does not allow the definition of generally accepted reference values but describes exploratory ranges.

## Conclusions

The results of our observational and cross-sectional study describe exploratory reference values for NT-proBNP in preterm infants ≤ 31 weeks GA in the early postnatal life. NT-proBNP levels during the first week of life are high and widely distributed in preterm infants and decrease subsequently to reach a distinctly lower and stable plateau at around 1 month of life. Our results suggest an influence of GA on NT-proBNP values in the first week of life.

## Supplementary information

ESM 1 (DOCX 16 kb)

ESM 2 (DOCX 16 kb)

ESM 3 (DOCX 16 kb)

ESM 4 (DOCX 24 kb)

ESM 5 (DOCX 25 kb)

ESM 6 (DOCX 26 kb)

ESM 7 (DOCX 26 kb)

ESM 8 (DOCX 49 kb)

ESM 9 (DOCX 25 kb)

ESM 10 (DOCX 25 kb)

ESM 11 (DOCX 22 kb)

## Data Availability

Derived data supporting the findings of this study are available from the corresponding author upon request.

## Abbreviations

BNP: Brain natriuretic peptide
BPD: Bronchopulmonary dysplasia
CRP: C-reactive protein
GA: Gestational age
hsPDA: Hemodynamically significant patent ductus arteriosus
IQR: Interquartile range
IVH: Intraventricular hemorrhage
La:Ao: Left atrial to aortic root ratio
NICU: Neonatal Intensive Care Unit
NT-proBNP: N-terminal pro-brain natriuretic peptide
PH: Pulmonary hypertension
ROP: Retinopathy of prematurity
SD: Standard deviation
WHO: World Health Organization
