# Supplementary material for: Reference values for N-terminal Pro-brain natriuretic peptide in premature infants during their first weeks of life
Source: Eur J Pediatr. 2020 Nov 3;180(4):1193–201. doi: 10.1007/s00431-020-03853-8 (PMC7940151; doi:10.1007/s00431-020-03853-8)
Supplement: Supplementary file 1 — (DOCX 16 kb) [file 431_2020_3853_MOESM1_ESM.docx]

| **Sampling time** | **n** | **Median** | **Mean** | **SD** | **Minimum** | **Maximum** | **IQR** |
| --- | --- | --- | --- | --- | --- | --- | --- |
| First week of life | 27 | 1,896 | 3,392 | 3,278 | 350 | 12,762 | 1,277-5,200 |
| 4±1 weeks of life | 26 | 463 | 515 | 236 | 199 | 1,101 | 364-704 |
| Corrected GA of 36±2 weeks of life | 33 | 824 | 991 | 539 | 148 | 2,531 | 714-1,232 |

**Table 2** NT-proBNP values of preterm infants ≤31 weeks GA without relevant complications over the first weeks of life
