# Supplementary material for: Reference values for N-terminal Pro-brain natriuretic peptide in premature infants during their first weeks of life
Source: Eur J Pediatr. 2020 Nov 3;180(4):1193–201. doi: 10.1007/s00431-020-03853-8 (PMC7940151; doi:10.1007/s00431-020-03853-8)
Supplement: Supplementary file 2 — (DOCX 16 kb) [file 431_2020_3853_MOESM2_ESM.docx]

| **Sampling time** | **n** | **Median** | **Mean** | **SD** | **Minimum** | **Maximum** | **IQR** |
| --- | --- | --- | --- | --- | --- | --- | --- |
| First week of life | 34 | 4,058 | 8,914 | 10,5431 | 557 | 39,340 | 2,189-10,751 |
| 4±1 weeks of life | 45 | 920 | 1,283 | 983 | 216 | 4,616 | 601-1,630 |
| Corrected GA of 36±2 weeks of life | 34 | 671 | 740 | 396 | 232 | 1,815 | 449-989 |

**Table 3** NT-proBNP values of preterm infants ≤31 weeks GA with relevant complications over the first weeks of life
