# Supplementary material for: Reference values for N-terminal Pro-brain natriuretic peptide in premature infants during their first weeks of life
Source: Eur J Pediatr. 2020 Nov 3;180(4):1193–201. doi: 10.1007/s00431-020-03853-8 (PMC7940151; doi:10.1007/s00431-020-03853-8)
Supplement: Supplementary file 3 — (DOCX 16 kb) [file 431_2020_3853_MOESM3_ESM.docx]

| **Sampling time** | **n** | **Median** | **Mean** | **SD** | **Minimum** | **Maximum** | **IQR** |
| --- | --- | --- | --- | --- | --- | --- | --- |
| First week of life | 61 | 3,264 | 6,470 | 8,570 | 350 | 39,340 | 1,573-8,038 |
| 4±1 weeks of life | 71 | 724 | 1,002 | 875 | 199 | 4,616 | 438-1,171 |
| Corrected GA of 36±2 weeks of life | 67 | 776 | 864 | 485 | 148 | 2,531 | 491-1,036 |

**Table 4** NT-proBNP values of preterm infants ≤31 weeks GA over the first weeks of life
