# Supplementary material for: Reference values for N-terminal Pro-brain natriuretic peptide in premature infants during their first weeks of life
Source: Eur J Pediatr. 2020 Nov 3;180(4):1193–201. doi: 10.1007/s00431-020-03853-8 (PMC7940151; doi:10.1007/s00431-020-03853-8)
Supplement: Supplementary file 4 — (DOCX 24 kb) [file 431_2020_3853_MOESM4_ESM.docx]

**Table 5** NT-proBNP levels in preterm infants ≤31 weeks GA without PDA or nhsPDA

| **Sampling time** | **n** | **Mean** | **Median** | **SD** | **Minimum** | **Maximum** | **IQR** |
| --- | --- | --- | --- | --- | --- | --- | --- |
| First week of life | 53 | 5,925 | 2,358 | 8,378 | 350 | 39,340 | 1,506-6,826 |
| 4±1 weeks of life | 59 | 1,018 | 704 | 939 | 199 | 4,616 | 438-1,101 |
| 36±2 weeks corrected GA | 59 | 906 | 799 | 493 | 148 | 2,531 | 644-1,093 |

**Table 6** NT-proBNP levels in preterm infants ≤31 weeks GA with hsPDA

| **Sampling time** | **n** | **Mean** | **Median** | **SD** | **Minimum** | **Maximum** | **IQR** |
| --- | --- | --- | --- | --- | --- | --- | --- |
| First week of life | 8 | 10,080 | 7,843 | 9,537 | 2,533 | 31,071 | 2,915-14,116 |
| 4±1 weeks of life | 12 | 923 | 1,024 | 464 | 216 | 1,610 | 421-1,278 |
| 36±2 weeks corrected GA | 8 | 551 | 470 | 286 | 232 | 1,036 | 321-827 |

**Table 7** Comparison of NT-proBNP levels between infants without PDA or nhsPDA and with hsPDA at the different sampling times using Mann-Whitney-U test

| **Sampling time** | **p-value obtained in Mann-Whitney-U test** | **Statistical dominance** |
| --- | --- | --- |
| First week of life | 0.034 | hsPDA |
| 4±1 weeks of life | 0.514 | hsPDA |
| 36±2 weeks corrected GA | 0.028 | no PDA or nhsPDA |

**Fig.4** Nomograms showing the 25^th^ percentile, 50^th^ and 75^th^ percentile for NT-proBNP values in ng/l in preterm neonates born ≤31 weeks GA over the first weeks of life. NT-proBNP for preterm infants without PDA or nhsPDA are presented on the left side, with hsPDA on the right side.
