# Supplementary material for: Reference values for N-terminal Pro-brain natriuretic peptide in premature infants during their first weeks of life
Source: Eur J Pediatr. 2020 Nov 3;180(4):1193–201. doi: 10.1007/s00431-020-03853-8 (PMC7940151; doi:10.1007/s00431-020-03853-8)
Supplement: Supplementary file 5 — (DOCX 25 kb) [file 431_2020_3853_MOESM5_ESM.docx]

**Table 8** NT-proBNP levels in preterm infants ≤31 weeks GA without BPD

| **Sampling time** | **n** | **Mean** | **Median** | **SD** | **Minimum** | **Maximum** | **IQR** |
| --- | --- | --- | --- | --- | --- | --- | --- |
| First week of life | 44 | 5,597 | 2,048 | 7,524 | 350 | 33,783 | 1,346-7,816 |
| 4±1 weeks of life | 45 | 632 | 511 | 346 | 199 | 1,457 | 396-778 |
| 36±2 weeks corrected GA | 51 | 935 | 799 | 518 | 148 | 2,531 | 665-1,211 |

**Table 9** NT-proBNP levels in preterm infants ≤31 weeks GA with BPD

| **Sampling time** | **n** | **Mean** | **Median** | **SD** | **Minimum** | **Maximum** | **IQR** |
| --- | --- | --- | --- | --- | --- | --- | --- |
| First week of life | 17 | 8,730 | 4,780 | 10,760 | 1,796 | 39,340 | 3,050-9,037 |
| 4±1 weeks of life | 26 | 1,643 | 1,186 | 1,122 | 413 | 4,616 | 813-2,634 |
| 36±2 weeks corrected GA | 16 | 637 | 656 | 265 | 254 | 1,013 | 408-879 |

**Table 10** Comparison of NT-proBNP levels between infants without and with BPD at the different sampling times using Mann-Whitney-U test

| **Sampling time** | **p-value obtained in Mann-Whitney-U test** | **Statistical dominance** |
| --- | --- | --- |
| First week of life | 0.021 | with BPD |
| 4±1 weeks of life | <0.001 | with BPD |
| 36±2 weeks corrected GA | 0.042 | without BPD |

**Fig.5** Nomograms showing the 25^th^ percentile, 50^th^ and 75^th^ percentile for NT-proBNP values in ng/l in preterm neonates born <31 weeks GA over the first weeks of life. NT-proBNP for preterm infants without BPD are presented on the left side, with BPD on the right side.
