# Supplementary material for: Reference values for N-terminal Pro-brain natriuretic peptide in premature infants during their first weeks of life
Source: Eur J Pediatr. 2020 Nov 3;180(4):1193–201. doi: 10.1007/s00431-020-03853-8 (PMC7940151; doi:10.1007/s00431-020-03853-8)
Supplement: Supplementary file 6 — (DOCX 26 kb) [file 431_2020_3853_MOESM6_ESM.docx]

**Table 11** NT-proBNP levels in preterm infants ≤31 weeks GA without PH

| **Sampling time** | **n** | **Mean** | **Median** | **SD** | **Minimum** | **Maximum** | **IQR** |
| --- | --- | --- | --- | --- | --- | --- | --- |
| First week of life | 48 | 5,660 | 2,808 | 7,343 | 350 | 33,783 | 1,498-7,816 |
| 4±1 weeks of life | 50 | 729 | 539 | 531 | 199 | 2,763 | 406-954 |
| 36±2 weeks corrected GA | 58 | 901 | 811 | 503 | 148 | 2,531 | 549-1,120 |

**Table 12** NT-proBNP levels in preterm infants ≤31 weeks GA with PH

| **Sampling time** | **n** | **Mean** | **Median** | **SD** | **Minimum** | **Maximum** | **IQR** |
| --- | --- | --- | --- | --- | --- | --- | --- |
| First week of life | 13 | 9,459 | 4,780 | 11,989 | 989 | 39,340 | 2,289-10,590 |
| 4±1 weeks of life | 21 | 1,653 | 1,171 | 1,165 | 496 | 4,616 | 810-2,645 |
| 36±2 weeks corrected GA | 9 | 618 | 674 | 246 | 254 | 988 | 409-817 |

**Table 13** Comparison of NT-proBNP levels between infants without PH and with PH at the different sampling times using Mann-Whitney-U test

| **Sampling time** | **p-value obtained in Mann-Whitney-U test** | **Statistical dominance** |
| --- | --- | --- |
| First week of life | 0.121 | PH |
| 4±1 weeks of life | <0.001 | PH |
| 36±2 weeks corrected GA | 0.087 | noPH |

**Fig. 6** Nomograms showing the 25^th^ percentile, 50^th^ and 75^th^ percentile for NT-proBNP values in ng/l in preterm neonates born <31 weeks GA over the first weeks of life. NT-proBNP for preterm infants without PH are presented on the left side, NT-proBNP for preterm infants with PH on the right side.
