# Supplementary material for: Reference values for N-terminal Pro-brain natriuretic peptide in premature infants during their first weeks of life
Source: Eur J Pediatr. 2020 Nov 3;180(4):1193–201. doi: 10.1007/s00431-020-03853-8 (PMC7940151; doi:10.1007/s00431-020-03853-8)
Supplement: Supplementary file 7 — (DOCX 26 kb) [file 431_2020_3853_MOESM7_ESM.docx]

**Table 14** NT-proBNP levels in preterm infants ≤31 weeks GA with ROP Stage 2 without Plus disease or lower

| **Sampling time** | **n** | **Mean** | **Median** | **SD** | **Minimum** | **Maximum** | **IQR** |
| --- | --- | --- | --- | --- | --- | --- | --- |
| First week of life | 57 | 5,979 | 3,264 | 7,641 | 350 | 33,783 | 1,545-7,714 |
| 4±1 weeks of life | 67 | 889 | 704 | 680 | 199 | 3,335 | 419-1,101 |
| 36±2 weeks corrected GA | 63 | 864 | 776 | 479 | 148 | 2,531 | 500-1,036 |

**Table 15** NT-proBNP levels in preterm infants ≤31 weeks GA with ROP Stage 2 with Plus disease or higher

| **Sampling time** | **n** | **Mean** | **Median** | **SD** | **Minimum** | **Maximum** | **IQR** |
| --- | --- | --- | --- | --- | --- | --- | --- |
| First week of life | 4 | 13,462 | 6,144 | 17,586 | 2,219 | 39,340 | 2,323-31,919 |
| 4±1 weeks of life | 4 | 2,888 | 3,095 | 1,635 | 748 | 4,616 | 1,227-4,343 |
| 36±2 weeks corrected GA | 4 | 863 | 645 | 664 | 367 | 1,795 | 377-1,567 |

**Table 16** Comparison of NT-proBNP levels between infants with ROP Stage 2 without Plus disease or lower and with ROP Stage 2 with Plus disease or higher at the different sampling times using Mann-Whitney-U test

| **Sampling time** | **p-value obtained in Mann-Whitney-U test** | **Statistical dominance** |
| --- | --- | --- |
| First week of life | 0.237 | ROP higher stages |
| 4±1 weeks of life | 0.007 | ROP higher stages |
| 36±2 weeks corrected GA | 0.808 | ROP lower stages |

**Fig. 7** Nomograms showing the 25^th^ percentile, 50^th^ and 75^th^ percentile for NT-proBNP values in ng/l in preterm neonates born ≤31 weeks GA over the first weeks of life. NT-proBNP for preterm infants with ROP Stage 2 without Plus disease or lower are presented on the left side, NT-proBNP for preterm infants with ROP Stage 2 with Plus disease or higher on the right side.
