# Supplementary material for: Reference values for N-terminal Pro-brain natriuretic peptide in premature infants during their first weeks of life
Source: Eur J Pediatr. 2020 Nov 3;180(4):1193–201. doi: 10.1007/s00431-020-03853-8 (PMC7940151; doi:10.1007/s00431-020-03853-8)
Supplement: Supplementary file 8 — (DOCX 49 kb) [file 431_2020_3853_MOESM8_ESM.docx]

**Table 17** NT-proBNP levels in preterm infants ≤31 weeks GA without infection

| **Sampling time** | **n** | **Mean** | **Median** | **SD** | **Minimum** | **Maximum** | **IQR** |
| --- | --- | --- | --- | --- | --- | --- | --- |
| First week of life | 38 | 4,380 | 2,058 | 4,662 | 350 | 21,217 | 1,327-6,423 |
| 4±1 weeks of life | 47 | 852 | 640 | 712 | 199 | 3,335 | 413-967 |
| 36±2 weeks corrected GA | 53 | 874 | 771 | 513 | 148 | 2,531 | 484-1,128 |

**Table 18** NT-proBNP levels in preterm infants ≤31 weeks GA with infection

| **Sampling time** | **n** | **Mean** | **Median** | **SD** | **Minimum** | **Maximum** | **IQR** |
| --- | --- | --- | --- | --- | --- | --- | --- |
| First week of life | 23 | 9,923 | 3,765 | 11,989 | 1,091 | 39,340 | 2,219-10,909 |
| 4±1 weeks of life | 24 | 1,296 | 1,003 | 1,086 | 260 | 4,616 | 531-1,542 |
| 36±2 weeks corrected GA | 14 | 824 | 830 | 374 | 313 | 1,815 | 546-997 |

**Table 19** Comparison of NT-proBNP levels between infants without infection and with infection at the different sampling times using Mann-Whitney-U test

| **Sampling time** | **p-value obtained in Mann-Whitney-U test** | **Statistical dominance** |
| --- | --- | --- |
| First week of life | 0.020 | infection |
| 4±1 weeks of life | 0.032 | infection |
| 36±2 weeks corrected GA | 0.939 | infection |

**Fig. 8** Nomograms showing the 25^th^ percentile, 50^th^ and 75^th^ percentile for NT-proBNP values in ng/l in preterm neonates born ≤31 weeks GA over the first weeks of life. NT-proBNP for preterm infants without infection are presented on the left side, with infection on the right side.
