# Supplementary material for: Reference values for N-terminal Pro-brain natriuretic peptide in premature infants during their first weeks of life
Source: Eur J Pediatr. 2020 Nov 3;180(4):1193–201. doi: 10.1007/s00431-020-03853-8 (PMC7940151; doi:10.1007/s00431-020-03853-8)
Supplement: Supplementary file 9 — (DOCX 25 kb) [file 431_2020_3853_MOESM9_ESM.docx]

**Table 20** NT-proBNP levels in preterm infants ≤31 weeks GA with IVH maximum Grade 1

| **Sampling time** | **n** | **Mean** | **Median** | **SD** | **Minimum** | **Maximum** | **IQR** |
| --- | --- | --- | --- | --- | --- | --- | --- |
| First week of life | 52 | 5,470 | 2,289 | 7,404 | 350 | 39,340 | 1,498-7,449 |
| 4±1 weeks of life | 64 | 855 | 677 | 672 | 199 | 3,524 | 415-1,074 |
| 36±2 weeks corrected GA | 61 | 859 | 769 | 500 | 148 | 2,531 | 490-1,046 |

**Table 21** NT-proBNP levels in preterm infants ≤31 weeks GA with IVH Grade 2 or higher

| **Sampling time** | **n** | **Mean** | **Median** | **SD** | **Minimum** | **Maximum** | **IQR** |
| --- | --- | --- | --- | --- | --- | --- | --- |
| First week of life | 9 | 12,248 | 5,508 | 12,540 | 2,634 | 33,783 | 3,611-23,019 |
| 4±1 weeks of life | 7 | 2,350 | 2,446 | 1,358 | 916 | 4,616 | 1,089-3,335 |
| 36±2 weeks corrected GA | 6 | 908 | 937 | 325 | 367 | 1,367 | 711-1,102 |

**Table 22** Comparison of NT-proBNP levels between infants without IVH maximum Grade 1 and with IVH Grade 2 or higher at the different sampling times using Mann-Whitney-U test

| **Sampling time** | **p-value obtained in Mann-Whitney-U test** | **Statistical dominance** |
| --- | --- | --- |
| First week of life | 0.014 | IVH Grade 2 or higher |
| 4±1 weeks of life | 0.001 | IVH Grade 2 or higher |
| 36±2 weeks corrected GA | 0.326 | IVH Grade 2 or higher |

**Fig. 9** Nomograms showing the 25^th^ percentile, 50^th^ and 75^th^ percentile for NT-proBNP values in ng/l in preterm neonates born ≤31 weeks GA over the first weeks of life. NT-proBNP for preterm infants with IVH maximum Grade 1 are presented on the left side, with IVH Grade 2 or higher on the right side.
