# Supplementary material for: Reference values for N-terminal Pro-brain natriuretic peptide in premature infants during their first weeks of life
Source: Eur J Pediatr. 2020 Nov 3;180(4):1193–201. doi: 10.1007/s00431-020-03853-8 (PMC7940151; doi:10.1007/s00431-020-03853-8)
Supplement: Supplementary file 10 — (DOCX 25 kb) [file 431_2020_3853_MOESM10_ESM.docx]

**Table 23** NT-proBNP levels in preterm infants ≤31 weeks GA without pulmonary complications

| **Sampling time** | **n** | **Mean** | **Median** | **SD** | **Minimum** | **Maximum** | **IQR** |
| --- | --- | --- | --- | --- | --- | --- | --- |
| First week of life | 55 | 6,084 | 3,264 | 8,038 | 350 | 39,340 | 1,578-7,918 |
| 4±1 weeks of life | 66 | 974 | 705 | 856 | 199 | 4,616 | 433-1,141 |
| 36±2 weeks corrected GA | 63 | 862 | 771 | 497 | 148 | 2,531 | 488-1,036 |

**Table 24** NT-proBNP levels in preterm infants ≤31 weeks GA with pulmonary complications

| **Sampling time** | **N** | **Mean** | **Median** | **SD** | **Minimum** | **Maximum** | **IQR** |
| --- | --- | --- | --- | --- | --- | --- | --- |
| First week of life | 6 | 10,009 | 2,929 | 12,921 | 557 | 31,071 | 1,153-23,681 |
| 4±1 weeks of life | 5 | 1,378 | 1,171 | 1,138 | 410 | 3,335 | 608-2,253 |
| 36±2 weeks corrected GA | 4 | 885 | 888 | 272 | 565 | 1,199 | 621-1,146 |

**Table 25** Comparison of NT-proBNP levels between infants without pulmonary complications and with pulmonary complications at the different sampling times using Mann-Whitney-U test

| **Sampling time** | **p-value obtained in Mann-Whitney-U test** | **Statistical dominance** |
| --- | --- | --- |
| First week of life | 0.934 | With pulmonary complications |
| 4±1 weeks of life | 0.275 | With pulmonary complications |
| 36±2 weeks corrected GA | 0.681 | With pulmonary complications |

**Fig.** **10** Nomograms showing the 25^th^ percentile, 50^th^ and 75^th^ percentile for NT-proBNP values in ng/l in preterm neonates born ≤31 weeks GA over the first weeks of life. NT-proBNP for preterm infants without pulmonary complications are presented on the left side, with pulmonary complications on the right side.
