# Supplementary material for: Reference values for N-terminal Pro-brain natriuretic peptide in premature infants during their first weeks of life
Source: Eur J Pediatr. 2020 Nov 3;180(4):1193–201. doi: 10.1007/s00431-020-03853-8 (PMC7940151; doi:10.1007/s00431-020-03853-8)
Supplement: Supplementary file 11 — (DOCX 22 kb) [file 431_2020_3853_MOESM11_ESM.docx]

**Table 26** NT-proBNP levels in preterm infants ≤31 weeks GA without intestinal complications

| **Sampling time** | **n** | **Mean** | **Median** | **SD** | **Minimum** | **Maximum** | **IQR** |
| --- | --- | --- | --- | --- | --- | --- | --- |
| First week of life | 53 | 5,458 | 2,533 | 6,902 | 350 | 33,783 | 1,506-7,714 |
| 4±1 weeks of life | 63 | 855 | 699 | 648 | 199 | 3,335 | 419-1,080 |
| 36±2 weeks corrected GA | 62 | 883 | 782 | 484 | 148 | 2,531 | 549-1,041 |

**Table 27** NT-proBNP levels in preterm infants ≤31 weeks GA with intestinal complications

| **Sampling time** | **n** | **Mean** | **Median** | **SD** | **Minimum** | **Maximum** | **IQR** |
| --- | --- | --- | --- | --- | --- | --- | --- |
| First week of life | 8 | 13,174 | 5,136 | 14,715 | 2,219 | 39,340 | 3,758-28,148 |
| 4±1 weeks of life | 8 | 2,157 | 1,953 | 1,491 | 305 | 4,616 | 959-3,368 |
| 36±2 weeks corrected GA | 5 | 620 | 343 | 476 | 254 | 1,367 | 284-1,096 |

**Table 28** Comparison of NT-proBNP levels between infants without intestinal complications and with intestinal complications at the different sampling times using Mann-Whitney-U test

| **Sampling time** | **p-value obtained in Mann-Whitney-U test** | **Statistical dominance** |
| --- | --- | --- |
| First week of life | 0.029 | Intestinal complications |
| 4±1 weeks of life | 0.007 | Intestinal complications |
| 36±2 weeks corrected GA | 0.200 | Without intestinal complications |

**Fig. 11** Nomograms showing the 25^th^ percentile, 50^th^ and 75^th^ percentile for NT-proBNP values in ng/l in preterm neonates born ≤31 weeks GA over the first weeks of life. NT-proBNP for preterm infants without intestinal complications are presented on the left side, with intestinal complications on the right side.
